# Extra-cellular vesicles of the male genital tract: new actors in male fertility?

**DOI:** 10.1186/s12610-021-00141-9

**Published:** 2021-10-14

**Authors:** Anne-Sophie Neyroud, Régina Chiechio, Marina Yefimova, Maria Josè Lo Faro, Nathalie Dejucq-Rainsford, Sylvie Jaillard, Pascale Even-Hernandez, Valérie Marchi, Célia Ravel

**Affiliations:** 1 CHU Rennes, Service de Biologie de la Reproduction-CECOS, 35000 Rennes, France; 2 Univ Rennes, Inserm, EHESP, Irset (Institut de recherche en santé, environnement et travail) - UMR_S 1085, F-35000 Rennes, France; 3 Physics and Astronomy Department “E. Majorana”, University of Catania, Via S. Sofia 64, 95123 Catania, Italy; 4 Univ Rennes, CNRS, ISCR (Institut des Sciences Chimiques de Rennes) - UMR 6226, F-35000 Rennes, France; 5 Sechenov Institute of Evolutionary Physiology and Biochemistry RAS, St-Petersburg, 194223 Russia

**Keywords:** Extra-cellular vesicles, Male genital tract, Prostasomes, Epididymosomes, Myelinosomes

## Abstract

Extracellular Vesicles (EVs) are membrane-limited particles containing proteins, lipids, metabolites and nucleic acids that are secreted by healthy and cancerous cells. These vesicles are very heterogeneous in size and content and mediate a variety of biological functions. Three subtypes of EV have been described in the male genital tract: microvesicles, myelinosomes and exosomes. Each type of EVs depends on the location of secretion such as the testis, prostate or epididymis. It has been shown that EVs can fuse together and deliver information to recipient cells, for example spermatozoa in the male genital tract. Cryo-electron microscopy remains the reference technique for determining EV morphology, but quantifying the absolute concentration of these EVs in biological fluids remains a challenge from a clinical point of view. The field of bio detection has considerably increased with the introduction of nanomaterials in biosensors and will provide a better understanding of the impact of these EVs. However, functional modifications of male gametes result from interactions with the components of the intraluminal fluid all along the genital tract and depend on the secretion and absorption of proteins and lipids from the local microenvironment. We cannot therefore exclude the possibility of epigenetic modulation of the information that will be transmitted to the embryo and therefore to the next generation via EVs.

## Résumé

Les Vésicules Extracellulaires (VE) sont des constituants d’origine membranaire contenant des protéines solubles ou membranaires, des lipides, des métabolites ou des acides nucléiques. Ces vésicules sont très hétérogènes en taille et en contenu. Trois catégories de VE ont été décrites dans le tractus génital mâle: les microvésicules, les myélinosomes et les exosomes. Les types de VE sont différents selon le lieu de production testiculaire, prostatique ou encore épididymaire. Il a été montré que les VE peuvent fusionner et délivrer une information à la cellule réceptrice, en l’occurrence le spermatozoïde dans le tractus génital mâle. La cryo-microscopie électronique reste la technique de référence pour déterminer la morphologie des VE mais la quantification de la concentration absolue de ces VE dans les liquides biologiques reste un challenge dans le cadre d’une approche clinique. Le domaine de la biodétection s’est. considérablement développé avec l’introduction des nanomatériaux dans les biocapteurs et va permettre de mieux comprendre l’impact de ces VE. Or les modifications fonctionnelles des gamètes mâles résultent d’interactions avec les composants du liquide intraluminal tout le long du tractus génital et dépendent de la sécrétion et de l’absorption de protéines et de lipides du microenvironnement local. On ne peut donc pas exclure la possibilité d’une modulation épigénétique des informations qui seront transmises à l’embryon donc à la génération suivante via les VE.

## Introduction

Extracellular vesicles (EVs) are membrane limited particles containing proteins, lipids, metabolites and nucleic acids that are secreted by healthy and cancerous cells. These vesicles are very heterogeneous in size and content and mediate a variety of biological functions. EVs are derived from cell membranes released into the extracellular medium. They are present in body’s fluids and may transmit information between cells [1]. Depending on their subcellular origin, EVs have specific signatures allowing them to be distinguished such as cytoskeleton proteins, Major Histocompatibility Complex proteins, chaperones, nucleic acid binding proteins or proteins involved in the membrane trafficking [2]. It is a heterogeneous population that can vary in size from 30 to 1000 nm [3]. The presence of EVs in human seminal plasma suggests a biological effect on human fertility. In the testis, Sertoli cells of the seminiferous tube secrete extracellular vesicles with a characteristic membrane coil structure called myelinosomes [4]. In the genital tract, extracellular vesicles are secreted by the epididymis (epididymosomes) and prostate (prostasomes) and play an important role in the maturation and capacitation of sperm, through changes in their membrane and in their metabolism. They allow the transfer of information to recipient cells.

The aim of this review is to describe the different EVs which may participate in various aspects of male fertility as component of sperm environnement at different steps of the male genital tract.

### Morphological diversity of extracellular vesicles

EVs are delimited by a lipid bilayer delineating a varied content of soluble or membrane proteins, lipids, metabolites, and nucleic acids. Tissue-specific molecular mediators characteristic of the secretory cell can be found on both EV membranes and in EV content. Molecules on the surface of EVs promote interaction with other cells by molecular recognition with lipids and ligands on the surface of the recipient cell. These attractive and specific interactions can induce the EV adhesion, fusion and even internalization of the entire EV with the plasma membrane of the recipient cell [5]. EVs are easy to detect by conventional colloid and nanoparticle detection techniques [6]. The compartimentalized structure of the EVs as well as their size (diameter of 30 nm–1000 nm) allow their detection by light scattering either at the individual scale by Dark Field Optical Microscopy (Nano Tracking Analysis NTA) [7] or at the macroscopic scale by Dynamic Light Scattering (DLS) [8]. Transmission electron microscopy (TEM) complements these techniques, giving both the size and the precise morphology of EVs (at the nm scale), particularly their multilamellar character by cryo-TEM or cryofracture techniques. Electron microscopy remains the reference technique for determining EV morphology [9]. To caracterise the EVs, it is possible to harvestvesicles of identical density from a heterogeneous suspension by density gradient ultracentrifugation. Ultrafiltration with size exclusion chromatography for the largest EVs may also useful. An alternative technique is a capture by specific recognition of surface proteins of EVs (for example CD63, CD9 or CD81) with magnetic beads or surfaces expressing by monoclonal antibodies [10]. These techniques allow the detection, isolation and separation of EVs. It is then possible to distinguish three subpopulations of EVs. Three subtypes of EV are described: microvesicles (MV), myelinosomes and exosomes. Vesicles have been isolated from diverse body fluids, including blood (endothelial cells, blood platelets, or immune cells such as CD4 T lymphocytes, macrophages), bile, urine, saliva, breast milk, amniotic fluid, ascites fluid, cerebrospinal fluid and semen [11, 12].

#### Microvesicles

These are buds from the plasma membrane released into the pericellular space. These EVs generally have a diameter of 100 to 1000 nm [13]. Coming from the budding of the plasma membrane, their composition is similar to that of the plasma membrane but may vary depending on the cell of origin.

#### Myelinosomes

The first observation was described in 1989 [14]. The term “myelinosome” refers to the characteristic electron microscopic appearance of myelin and its markedly electron-dense osmiophilic membranes. In fact, myelinosomes appear as vesicles delimited by a wall of concentric lamellar structures, regularly ordered, consisting of the alternation of dense lines and clear bands reminiscent of myelin. These EVs have a diameter of 200–700 nm [4]. These myelinosomes are composed of two areas: an outer layer with a network of concentric osmiophilic membranes and an electron-transparent matrix. Rough endoplasmic reticulum cisterns are seemingly associated with their onset and development. These myelinosomes are membrane organelles identified in the seminiferous epithelium of the testis and secreted by Sertoli somatic cells in the lumen of the seminiferous tubules in transgenic R6/1 Huntington disease mice. It has been suggested that Sertoli cells remove the misfolded proteins through this myelinosome-mediated secretion, thus preserving cellular proteostasis. While rare in normal cells, intracellular myelinosomes appear in pathological situations caused by genetic or environmental factors [15]. They are also detected and abundant in aggregation diseases such as Huntington’s disease or cystic fibrosis [16]. Many of these electron-dense osmiophilic membranes are also found in lysosomal overload disease. Indeed myelinosomes are considered to act as storage for organelles along with autolysosomes, endolysosomes and late endosomes [17]. They differ from exosomes and microvesicles in their morphology and origin. Our recent study demonstrated the presence of myelinosome-like vesicles in human seminal plasma [4].

#### Exosomes

These small EVs have a diameter of 30 to 100 nm [18]. They are formed inside cells by budding late endosomes, called multivesicular bodies, and are then released into the extracellular environment by the fusion of these multivesicular bodies with the plasma membrane. Proteins commonly found on EVs membranes include tetraspanins, especially CD63, CD9, CD81, heat shock proteins (HSP70), and glycophosphatidylinositol anchored proteins.

### Particular properties of human seminal plasma

Seminal plasma represents the environment of male gametes that is very rich in EVs. Human seminal plasma is a complex fluid produced by secretions from several glands of the male genital tract (prostate, epididymis, seminal vesicles) and constitutes over 95% of semen volume. The remaining 10% of semen volume is made up of secretions from the Cowper (bulbo-urethral) and Littre glands [19].. Its sperm-free fluid may be analysed after liquefaction and centrifugation of the ejaculated semen. Owing to its viscosity and its water-holding capacity, conventional Transmission Electron Microscopy (TEM) is not optimal for analysing EVs. Cryoelectron microscopy (cryo-TEM) allows the visualisation of nanoscale structures without prior fixation or addition of heavy metals needed to enhance the medium contrast [20]. The sample is therefore visualised as closely as possible to its native physiological state, producing a projection through its three-dimensional sample volume [20]. In humans EVs are synthesized in particular by the prostate, as well as by the epididymis, and even by the testis. The structural heterogeneity and protein composition of these vesicles has been shown in human semen. EVs from seminal plasma participate in various aspects of male fertility, improving sperm function by regulating the timing of sperm capacitation, inducing acrosome reaction, stimulating sperm motility enabling them to reach the ovocyte. Other supposed functions of prostasomes include interference with immune cells of the female reproductive system. Indeed, it has been observed that fertility outcomes are significantly improved when women are exposed to seminal plasma [21].

Human seminal plasma particles were first classified according to morphological criteria, including the multivesicular aspect, the shape, the external protuberances and the density of the vesicles. In recent years, the development of new technologies in electron microscopy has led to an increase in knowledge with finer and more specific analysis for all types of EVs. A recent study extended the data revealing five major categories and six subcategories of extracellular membrane compartments, including lamellar bodies [22]. We have thus identified myelinosome-like vesicles in the seminal plasma of normozoospermic men by CryoTEM [4] however their biological role remains to be specified.

### Diversity of particulate location in the male genital tract

**Fig. 1:**
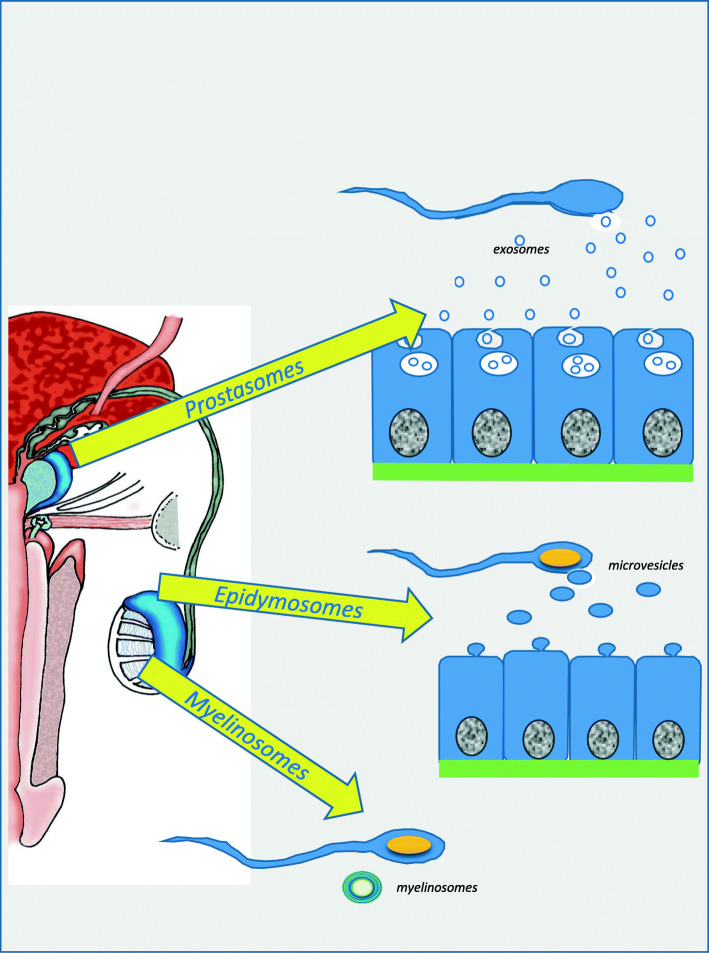
Extra-cellular Vesicles localization synthesis in the male genital tract

**Fig. 2:**
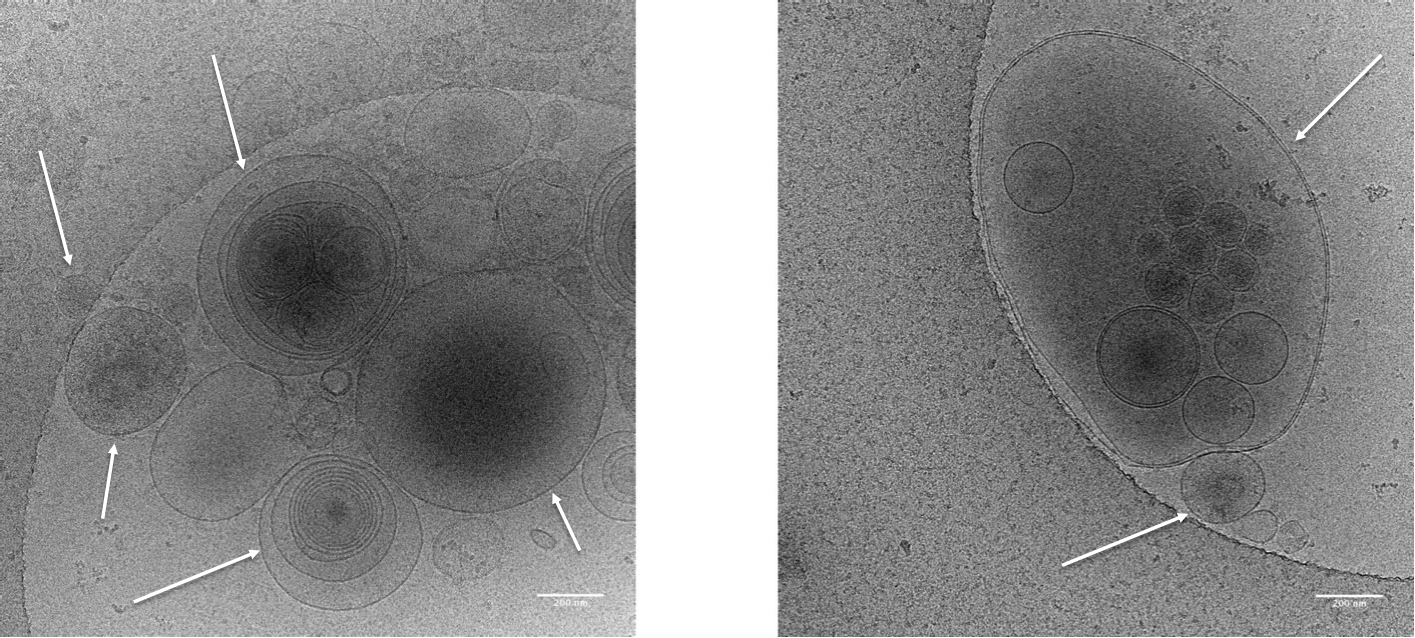
Analysis by CroTEM (Cryogenic Transmission Electron Microscopy) of human seminal plasma showing several EV (Extra-cellular Vesicles). Heterogeneity of EVs in seminal plasma

Different types of EV have been described according to their testicular, prostatic or epididymal localisation (Fig. 1). EVs have been shown to contain proteins, lipids (especially high levels of sphingomyelins), DNA and several varieties of RNA (Ribonucleic acid), including microRNAs (miRNAs) and mRNA fragments [23]. The characterisation of extracellular vesicles such as prostasomes in biological samples (seminal plasma) mainly requires the visualisation of these vesicles which can be accomplished either on the basis of their specific protein markers, or by transmission electron microscopy (TEM) or cryo-TEM (Fig. 2).

#### The testis

**Fig. 3:**
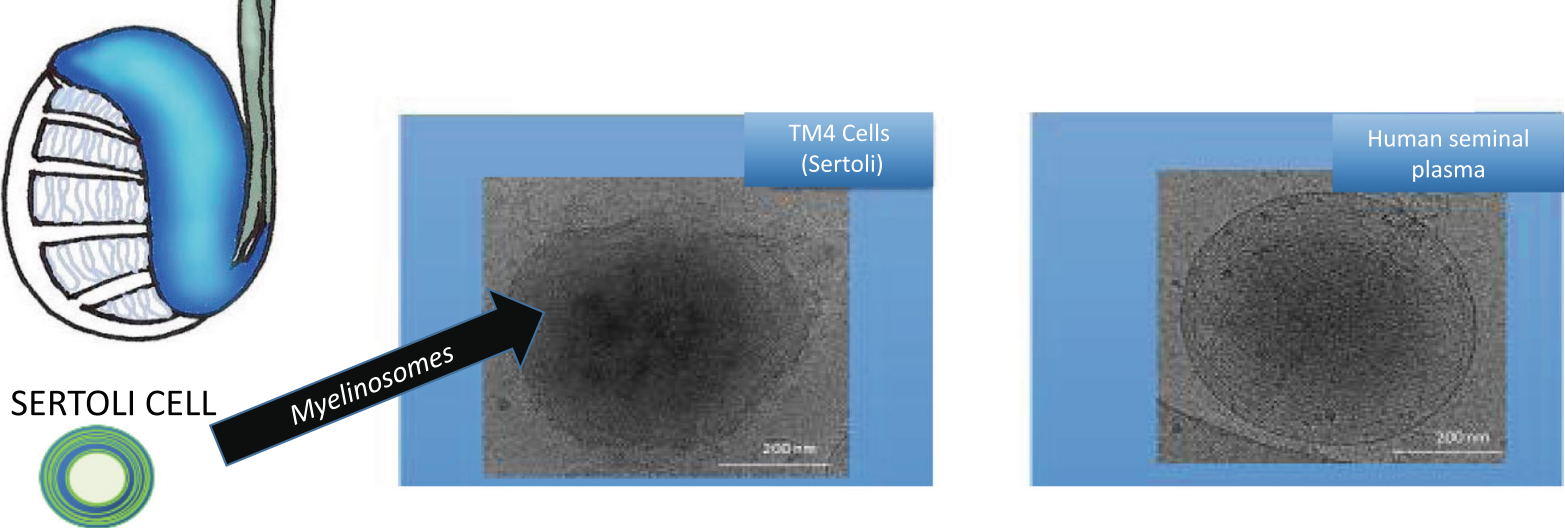
Comparative analysis of EVs (Extra-cellular Vesicles) from human seminal plasma and secretion products from TM4 sertoli cells. In preparations from both human seminal plasma (to the left) and culture media of TM4 Sertoli cells (to the right), myelinosomes vesicles were present together with other EVs. Myelinosomes round-shaped vesicles, formed with whorled membranes, exhibited strong electron density

Recently, Yefimova et al. demonstrated the secretion of myelinosomes by cultured Sertoli cells (TM4 cells) [16]. Myelinosomes are secretory organelles loaded with specific cargoes and capable of leaving the cell in their entirety, in the form of extracellular vesicles. This differentiates them from other intracellular secretory organelles, especially from the family of lysosome-related organelles (LRO: Lysosome-Related-Organelles), such as the secretory granules of blood immune cells, neurons, neuroendocrine cells, lamellar bodies, pulmonary or cutaneous keratinocytes, melanosomes or even secretory lysosomes [24]. All of them release their contents into the extracellular space after fusion with the plasma membrane, but not as intact organelles outside the cell. This mechanism is similar to that of LRO-type secretory granules from eosinophils, released in the form of organelles during a specific process linked to phagocytosis called NET (Neutrophil Extracellular Trap) [25]. The maturation process of myelinosomes in Sertoli cells resembles that of the lamellar bodies found in pulmonary epithelial cells. The lamellar bodies mature and traffic in Multi-Vesicular Bodies (MVBs) [26]. However, fusion of MVB with the plasma membrane results in the discharge of pulmonary surfactant, while at the testicular level, in Sertoli cells, mature myelinosomes are released in their entirety into extracellular spaces. In a transgenic R6/1 Huntington Disease mice, some myelinosomes are observed in the seminiferous epithelium of the testis. These myelinosomes are secreted by Sertoli’s somatic cells in the lumen of the seminiferous tubules [27]. Regarding the fate of myelinosomes released into extracellular spaces, we have shown the presence of myelinosome-like vesicles in human seminal fluid, but the function infertility is not yet understood [4]. The physiological role of myelinosomes in human seminal fluid remains to be established. We are only at the beginning of our knowledge of myelinosomes. To date, we only note their presence in human seminal plasma (Fig. 3), and future studies will provide information on their role and importance for human fertility.

#### The prostate

Prostasomes are nanostructured exosomes secreted by the acinar lumen of prostate epithelial cells. These prostasomes are 50 to 500 nm in size and contain proteins, lipids and nucleic acids. Despite the presence of merocrine secretory cells, the majority of secretory epithelial cells appear to be apocrine, with a wrapping of cells in human prostate stroma. The zinc levels detected in merocrine cells, but not in prostasomes, suggest that a significant proportion of prostasomes, possibly the majority, are generated via apocrine secretion. This finding provides an explanation why so many large proteins, without a signal peptide sequence, are present in the prostatic fluid [28]. Human prostasomes transport at least 440 proteins, including prostate-specific proteins (signal transduction proteins, chaperone proteins, GTP (Guanosine-5′-triphosphate) binding proteins, α proteins, structural proteins and transport proteins). It also contains genetic material such as RNA [29]. Prostasomes have many characteristics that differentiate them from other secretions of the prostate gland. They are rich in cholesterol and sphingomyelin, with a particularly high cholesterol / phospholipid ratio [30]. Sphingomyelin is the major component of the prostasome membrane and is thought to be protective against attack by reactive oxygen species (ROS). Polynuclear neutrophils (PNNs) secrete ROS, and prostasomes have the ability to decrease the production of ROS in semen containing PNNs. Indeed they facilitate the transfer of lipids to the plasma membrane of PNNs which inhibits the activity of Nicotinamide Adenine Dinucleotide Phosphate (NADPH) oxidase, thereby protecting spermatozoa from oxidative stress [31, 32]. Prostasomes play an active role in controlling capacitation and the acrosome reaction. In particular, prostasomes participate in preventing premature capacitation of spermatozoa and premature acrosome reaction. Indeed, the prostasomal membrane is enriched in cholesterol, which contributes to its stability in the acidic vaginal environment [33]. Owing to its high content of cholesterol and fatty acids in prostasomes, the plasma membrane of the sperm head stabilises and delays the occurrence of a premature acrosome reaction. Many biochemical and molecular markers can be identified in prostasomes that are useful for the diagnosis and prognosis of human male infertility. For example, a study have revealed significantly decreased seminal plasma Prostate-specific membrane antigen (PSMA) levels in patients with OAT syndrome. PSMA is a type II transmembrane glycoprotein predominantly expressed by the prostate epithelial cells. In several proteomic analyses, PSMA has consistently been reported to be present in seminal plasma and in extracellular vesicles and prostasomes isolated from the seminal plasma of both healthy and asthenozoospermic men. Although the precise role of folates in seminal plasma is not known, their higher concentration in semen compared to blood suggests that they might have other important functions for the sperm cells apart from their passive role as scavengers of reactive oxygen species in seminal plasma [34]. So, that is a molecular marker for human male infertility. An other example, histone H2B type 1-A (HIST1H2BA), a testis-specific histone variant with an essential role in sperm DNA organization and sperm histone–protamine transition, was overexpressed in prostasomes from non-normozoospermic men [35].

Prostasomes are also directly involved in immune regulation. Indeed, Natural killer (NK) cells, essential elements of innate immunity are abundant in the female reproductive system. Prostasomes decrease the activity of NK cells in the female genital tract, through the CD48 ligand of CD244 [20]. Prostasomes play a major role because they immunomodulate the local environment within the female reproductive system. This allows immune-mediated sperm protection until fertilisation. The antibacterial activity of human prostasomes has been well documented [36, 37]. It is established that prostasomes are very rich in calcium which plays a major role in sperm motility and the capacitation process. Sperm motility is promoted by ionic signals induced by calcium ions (Ca^2+^) through the region of the sperm midpiece. Prostasomes play a crucial role in maintaining long-lasting Ca^2 +^ signals in spermatozoa through the pH-dependent fusion between vesicles [31].

#### The epididymis

Spermatozoa must travel a long way through the epididymis from the testis, before reaching the vas deferens. The ability of spermatozoa to fertilise an ovocyte is gradually acquired through membrane changes throughout the epididymis, before interaction with seminal fluid at ejaculation, the female environment and the fusion with the mature oocyte. The epididymis has several functions, including the transport, maturation and storage of male gametes [38]. The epididymis is divided into three main segments in humans: head, body and tail. Each segment forms its own luminal microenvironment with different protein secretions and different cellular gene expression, optimised for each stage of sperm maturation [39]. The head and body are responsible for maturing spermatozoa, while the tail serves as a reservoir of spermatozoa [40, 41]. During their transit through the epididymis, their plasma membrane surface proteins undergo remodelling (i.e. changes in the composition of phospholipids with increased total negative charges and modification of surface proteins).

This maturation is dependent of the interaction between proteins and EVs secreted in the intraluminal fluid of the epididymis. Epididymosomes are EVs produced by epididymal epithelial cells by apocrine secretion [42], that is, the formation of cytoplasmic blebs at the apical pole of epithelial cells that detach in the lumen. They are 50 nm to 500 nm in diameter with a high cholesterol/phospholipid ratio and contain adhesion molecules such as tetraspanins, integrins and growth factors [43]. Studies in the bovine model have shown that epididymosomes contain several proteins that are involved in the acquisition of sperm mobility, fertilisation capacity and protection against oxidative stress [44]. The content of these vesicles also depends on the region of the epididymis and in particular some mi-RNAs are expressed to different extents according to the region [45].

### The potential transgenerational impact

Molecules on the surface of EVs promote interaction with other cells by adhesion of vesicles to lipids and ligands on their plasma membranes, internalisation of the entire vesicle or fusion of the EV membrane to the recipient cell. It has been shown that EVs can merge and deliver information to the receiving cell. In the male genital tract, the target of these EVs is the spermatozoon. We therefore cannot exclude the possibility of modulating the information that will be transmitted to the embryo and therefore to the next generation. The sperm RNA profile is now recognised as an important epigenetic player in the early development of the embryo and the health of the offspring [46]. Indeed, during epididymal transit a specific remodelling of the epigenetic profile occurs so that a spermatozoon is able not only to initiate fertilisation but also to maintain early embryonic development [45]. However, under stress conditions, the plasticity of the RNA profile has been demonstrated [47]. Among these mechanisms, extracellular vesicles such as epididymosomes or prostasomes are highlighted. These specialised extracellular vesicles are involved in a new form of intercellular communication, which is capable of dramatically altering the proteomic, lipidomic and epigenetic landscape of the maturing spermatozoa. Further research in this area promises to advance our understanding of this communication and the transgenerational transmission of epigenetic material.

### Biological EV analysis

In order to use EVs for diagnostic or therapeutic purposes, it is essential not only to detect them but also to quantify them at levels corresponding to those present in biological fluids [48, 49]. Their protein and genomic constituents can be detected by common techniques such as western blotting and genomic analysis (PCR) to caracterise them more finely [50].

On the other hand, quantifying the absolute concentration of exosomes in a liquid biopsy remains a challenge. Indeed, the single samples collected from patients are of very small volumes (a few tens of microliters) and require a very low level of detection of exosomes or their constituents (notably miRNA or proteins) since the idea is to make an early diagnosis of disease. The objective is to analyse these liquid biopsies in real time with a detection threshold of an interaction and concentration from the perspective of clinical medicine.

The specific detection of very low (femtoMolar) concentrations in real time remains a challenge as it is difficult to quantify EVs because of limitation of instruments used to investigate them.

Most of analyses combining fluorescence with an amplification system [51, 52] or high throughput microfluidic chips [53] have considerably contributed to improve the performance of biosensors. In particular, FRET (Fluorescence Resonance Energy Transfer) is a very accurate nanometer-scale measurement of the distance between two fluorescent probes due to a dipolar coupling between them. As it is effective only at a very short distance, it is possible to detect specifically a fluorescence interaction with very high sensitivity [54]. The introduction of nanomaterials has considerably increased the detection sensitivity and reliability of biosensors. Quantum dots (QDs), whose emission length can be modulated to the nanometer level, have been successfully used as fluorescent probes to quantify exosomes [55, 56]. They possess optical properties such as very high brightness (fluorescence quantum efficiency) and wide absorption band that are particularly well suited to multiplexed biosensing [57]. However, while their toxicity is not a real problem for in vitro diagnostics, their minimum size (around 10 nm) can be an obstacle when they are associated with a DNA amplification system. Metallic luminescent nanoclusters, especially gold [58] offer a promising compromise since their size is close to that of molecules (1–2 nm) and their fluorescence does not exhibits photobleaching [59].

## Conclusion

The heterogeneous population of EVs present in seminal plasma is known to influence these key changes associated with sperm functions [60]. The role of EVs in male reproduction has recently gained more attention and extensive research is focusing on their implications in male infertility, which may shed light on the potential role of new biomarkers different from those actually performed in Assisted Reproductive Technics laboratories [61]. Indeed, tissue regeneration relies on healing factors carried by EVs to generate a trophic microenvironment suitable for diseases. EVs can be untouched and used as therapeutic agents in their regular or natural state after isolation from biological samples or conditioned medium in standard cell culture conditions [62]. There is still a need for a consensus concerning EV isolation methods and EV characterization/designation remains. The potential therapeutic effects of EVs are still under consideration and more studies are needed to clarify their implication in male fertility.

## Data Availability

NA

## Abbreviations

Alix: ALG-2 interacting protein X
ART: Assisted Reproductive Technics
CD: Cluster of Differentiation
CryoTEM: Cryogenic Transmission Electron Microscopy
EV: Extra-cellular Vesicle
FRET: Fluorescence Resonance Energy Transfer
GTP: Guanosine-5′-triphosphate
HIV: Human Immunodeficiency Viruses
HSP70: 70 kd Heat Shock Proteins
LRO: Lysosome-Related-Organelles
MV: Micro Vesicles
MVB: Multi-Vesicular Bodies
NADPH: Nicotinamide Adenine Dinucleotide Phosphate
NET: Neutrophil Extracellular Trap or trapextracellular neutrophil
NK: Natural killer
PNN: Polynuclear neutrophils
TEM: Transmission Electron Microscopy
TM4: Murine Sertoli cell line
Tsg101: Tumor Susceptibility 101
RNA: Ribonucleic acid
ROS: Reactive Oxygen Species

## References

[1] Kowal J, Arras G, Colombo M, Jouve M, Morath JP, Primdal-Bengtson B (2016). Proteomic comparison defines novel markers to characterize heterogeneous populations of extracellular vesicle subtypes. Proc Natl AcadSci U S A.

[2] Le Lay S, Martinez MC, Andriantsitohaina R (2018). Extracellular vesicles as biomarkers and bioeffectors of metabolic syndrome. Med Sci MS.

[3] Carlos S, David W, David B, Nuria B, Lois AS, Felipe V (2018). Extracellular vesicles in human reproduction in health and disease. Endocr Rev.

[4] Yefimova M, Bere E, Neyroud AS, Jegou B, Bourmeyster N, Ravel C (2020). Myelinosome-like vesicles in human seminal plasma: a cryo-electron microscopy study. Cryobiology..

[5] Van Niel G, D’Angelo G, Raposo G (2018). Shedding light on the cell biology of extracellular vesicles. Nat Rev Mol Cell Biol.

[6] Xu L (2020). Optical, Electrochemical and Electrical (Nano) Biosensors for Detection of Exosomes: A Comprehensive Overview. Biosens Bioelectron.

[7] Shpacovitch V, Hergenroder R (2018). Optical and surface Plasmonic approaches to characterize extracellular vesicles. A Review. AnalChimActa.

[8] Thane KE, Davis AM, Hoffman AM (2019). Improved Methods for Fluorescent Labeling and Detection of Single Extracellular Vesicles Using Nanoparticle Tracking. Analysis Sci Rep.

[9] Coumans FAW, Brisson AR, Buzas EI, Dignat-George F, Drees EEE, El-Andaloussi S (2017). Methodological guidelines to study extracellular vesicles. Circ Res.

[10] Bachurski D, Schuldner M, Nguyen PH, Malz A, Reiners KS, Grenzi PC, Babatz F, Schauss AC, Hansen HP, Hallek M (2019). Pogge von Strandmann E. Extracellular Vesicle Measurements with Nanoparticle Tracking Analysis – An Accuracy and Repeatability Comparison between NanoSight NS300 and ZetaView. JExtracell Vesicles.

[11] Ismail N, Wang Y, Dakhlallah D, Moldovan L, Agarwal K, Batte K (2013). Macrophage microvesicles induce macrophage differentiation and miR-223 transfer. Blood..

[12] Raposo G, Stoorvogel W (2013). Extracellular vesicles: exosomes, microvesicles, and friends. J Cell Biol.

[13] Borges FT, Reis LA, Schor N (2013). Extracellular vesicles: structure, function, and potential clinical uses in renal diseases. Braz J Med Biol Res Rev Bras PesquiMedicas E Biol.

[14] Ultrastructural Pathology of the Cell and Matrix [Internet]. Elsevier. 1988 [13 août 2020]. Disponible sur: https://linkinghub.elsevier.com/retrieve/pii/C20130062470

[15] Yefimova M, Ravel C, Neyroud AS, Béré E, Bourmeyster N (2020). Myelinosomes: A new pathway of protein quality control. Med Sci (Paris).

[16] Yefimova MG, Béré E, Cantereau-Becq A, Harnois T, Meunier A-C, Messaddeq N (2016). Myelinosomes act as natural secretory organelles in Sertoli cells to prevent accumulation of aggregate-prone mutant huntingtin and CFTR. Hum Mol Genet.

[17] Yefimova MG, Bourmeyster N (2017). Myelinosome-driven secretion: non-catabolic management of misfolded proteins - lessons from the Sertoli cells. J Rare Dis Res Treat.

[18] Crescitelli R, Lässer C, Szabó TG, Kittel A, Eldh M, Dianzani I (2013). Distinct RNA profiles in subpopulations of extracellular vesicles: apoptotic bodies, microvesicles and exosomes. J Extracell Vesicles.

[19] Owen D, Katz D (2005). A review of the physical and chemical properties of human semen and the formulation of a semen simulant. J Androl.

[20] Stewart P (2017). Cryo-electron microscopy and cryo-electron tomography of nanoparticles. Methods Mol Biol.

[21] Tarazona R, Delgado E, Guarnizo MC, Roncero RG, Morgado S, Sánchez-Correa B (2011). Human prostasomes express CD48 and interfere with NK cell function. Immunobiology..

[22] Höög JL, Lötvall J (2015). Diversity of extracellular vesicles in human ejaculates revealed by cryo-electron microscopy. J Extracell Vesicles.

[23] Théry C, Ostrowski M, Segura E (2009). Membrane vesicles as conveyors of immune responses. Nat Rev Immunol.

[24] Marks MS, Heijnen HFG, Raposo G (2013). Lysosome-related organelles: unusual compartments become mainstream. CurrOpin Cell Biol.

[25] Delgado-Rizo V, Martínez-Guzmán MA, Iñiguez-Gutierrez L, García-Orozco A, Alvarado-Navarro A, Fafutis-Morris M (2017). Neutrophil extracellular traps and its implications in inflammation: an overview. Front Immunol.

[26] Whitsett JA, Weaver TE (2015). Alveolar development and disease. Am J Respir Cell Mol Biol.

[27] Yefimova M, Bourmeyster N, Becq F, Burel A, Lavault MT, Jouve G (2019). Update on the cellular and molecular aspects of cystic fibrosis transmembrane conductance regulator (CFTR) and male fertility. Morphologie..

[28] Fullwood NJ, Lawlor AJ, Martin-Hirsch PL, Matanhelia SS, Martin FL (2019). An analysis of benign human prostate offers insights into the mechanism of apocrine secretion and the origin of prostasomes. Sci Rep.

[29] Poliakov A, Spilman M, Dokland T, Amling CL, Mobley JA (2009). Structural heterogeneity and protein composition of exosome-like vesicles (prostasomes) in human semen. Prostate.

[30] Brouwers JF, Aalberts M, Jansen JWA, van Niel G, Wauben MH, Stout TAE (2013). Distinct lipid compositions of two types of human prostasomes. Proteomics..

[31] Burden HP, Holmes CH, Persad R, Whittington K (2006). Prostasomes--their effects on human male reproduction and fertility. Hum Reprod Update.

[32] García-Rodríguez A, Gosálvez J, Agarwal A, Roy R, Johnston S (2018). DNA Damage and Repair in Human Reproductive Cells. Int J Mol Sci.

[33] Pons-Rejraji H, Artonne C, Sion B, Brugnon F, Canis M, Janny L (2011). Prostasomes: inhibitors of capacitation and modulators of cellular signalling in human sperm. Int J Androl.

[34] Arslan MA, Avcı B, Tunçel ÖK, Asci R (2021). Decreased prostate-specific membrane antigen levels in the seminal plasma of oligoasthenoteratozoospermic men. Andrologia..

[35] García-Rodríguez A, de la Casa M, Peinado H, Gosálvez J, Roy R (2018). Human prostasomes from normozoospermic and non-normozoospermic men show a differential protein expression pattern. Andrology..

[36] Carlsson L, Påhlson C, Bergquist M, Ronquist G, Stridsberg M (2000). Antibacterial activity of human prostasomes. Prostate.

[37] Li J, Liu K, Liu Y, Xu Y, Zhang F, Yang H (2013). Exosomes mediate the cell-to-cell transmission of IFN-α-induced antiviral activity. Nat Immunol.

[38] Cooper TG (1998). Interactions between epididymal secretions and spermatozoa. J ReprodFertil Suppl.

[39] Dacheux J-L, Belleannée C, Guyonnet B, Labas V, Teixeira-Gomes A-P, Ecroyd H (2012). The contribution of proteomics to understanding epididymal maturation of mammalian spermatozoa. SystBiolReprod Med.

[40] Sullivan R (2016). Epididymosomes: role of extracellular microvesicles in sperm maturation. Front BiosciSch Ed.

[41] Sullivan R, Saez F (2013). Epididymosomes, prostasomes, and liposomes: their roles in mammalian male reproductive physiology. Reprod Camb Engl.

[42] Hermo L, Jacks D (2002). Nature’s ingenuity: bypassing the classical secretory route via apocrine secretion. Mol Reprod Dev.

[43] Thimon V, Frenette G, Saez F, Thabet M, Sullivan R (2008). Protein composition of human epididymosomes collected during surgical vasectomy reversal: a proteomic and genomic approach. Hum Reprod.

[44] Girouard J, Frenette G, Sullivan R (2011). Comparative proteome and lipid profiles of bovine epididymosomes collected in the intraluminal compartment of the caput and cauda epididymidis. Int J Androl.

[45] Conine CC, Sun F, Song L, Rivera-Pérez JA, Rando OJ (2018). Small RNAs gained during epididymal transit of sperm are essential for embryonic development in mice. Dev Cell.

[46] Rando OJ. Intergenerational Transfer of Epigenetic Information in Sperm. Cold SpringHarbPerspect Med [Internet]. 2016;6(5) [cité 14 août 2020], Disponible sur: https://www.ncbi.nlm.nih.gov/pmc/articles/PMC4852801/. 10.1101/cshperspect.a022988 PMC4852801 26801897

[47] Spadafora C (2018). The « evolutionary field » hypothesis. Non-Mendelian transgenerational inheritance mediates diversification and evolution. Prog Biophys Mol Biol.

[48] Théry C, Zitvogel L, Amigorena S (2002). Exosomes: composition, biogenesis and function. Nat Rev Immunol.

[49] Chapu N, Théry C (2011). Exosomes: immune properties and potential clinical implementations. Semin Immunopathol.

[50] Boriachek K, Islam MN, Gopalan V, Lam AK, Nguyen NT, Shiddiky MJA (2017). Quantum dot-based sensitive detection of disease specific exosome in serum. Analyst.

[51] Qiu X, Guo J, Xu J, Hildebrandt N (2018). Three-dimensional FRET multiplexing for DNA quantification with Attomolar detection limits. J Phys Chem Lett.

[52] Qiu X, Xu J, Guo J, Yahia-Ammar A, Kapetanakis NI, Duroux-Richard I (2018). Advanced MicroRNA-based Cancer diagnostics using amplified time-gated FRET. Chem Sci.

[53] Yang Q, Cheng L, Hu L, Lou D, Zhang T, Li J, Zhu Q, Liu F. An integrative microfluidic device for isolation and ultrasensitive detection of lung cancer-specific exosomes from patient urine. Biosens Bioelectron. 2020;163:112290. 10.1016/j.bios.2020.112290. 10.1016/j.bios.2020.112290 32568696

[54] Algar WR, Hildebrandt N, Vogel SS, Medintz IL (2019). FRET as a biomolecular research tool — understanding its potential while avoiding pitfalls. Nat Methods.

[55] Dobhal G, Ayupova D, Laufersky G, Ayed Z, Nann T, Goreham R (2018). Cadmium-free quantum dots as fluorescent labels for exosomes. Sensors.

[56] Bai Y, Lu Y, Wang K, Cheng Z, Qu Y, Qiu S, et al. Rapid isolation and multiplexed detection of exosome tumor markers via queued beads combined with quantum dots in a microarray. Nano Micro Letters. 2019;11. 10.1007/s40820-019-0285-x. 10.1007/s40820-019-0285-x PMC7770845 34137993

[57] Cardoso Dos Santos M, Algar WR, Medintz IL, Hildebrandt N (2020). Quantum Dots for Förster Resonance Energy Transfer (FRET). Trends Anal Chem.

[58] Alonso MC, Trapiella-Alfonso L, Fernández JMC, Pereiro R, Sanz-Medel A (2016). Functionalized gold nanoclusters as fluorescent labels for immunoassays: application to human serum immunoglobulin E determination. Biosens Bioelectron.

[59] Nonappa (2020). Luminescent Gold Nanoclusters for Bioimaging Applications. Beilstein J Nanotechnol.

[60] Machtinger R, Laurent LC, Baccarelli AA (2016). Extracellular vesicles: roles in gamete maturation, fertilization and embryo implantation. Hum Reprod Update.

[61] Hamdi SM, Sanchez E, Garimbay D, Albarede S (2020). External quality assessment program for biochemical assays of human seminal plasma: a French 6-years experience. Basic ClinAndrol.

[62] Velot É, Madry H, Venkatesan JK, Bianchi A, Cucchiarini M (2021). Is extracellular vesicle-based therapy the next answer for cartilage regeneration?. Front Bioeng Biotechnol.

